# Single-Bundle Versus Double-Bundle Arthroscopic Anterior Cruciate Ligament Reconstruction: Comparison of Long-Term Functional Outcomes

**DOI:** 10.7759/cureus.12243

**Published:** 2020-12-23

**Authors:** Vicknesh Anandan, Teik Chiang Goh, Kamarul Syariza Zamri

**Affiliations:** 1 Orthopaedics and Traumatology, Universiti Kebangsaan Malaysia Medical Centre, Kuala Lumpur, MYS

**Keywords:** anterior cruciate ligament (acl), arthroscopic reconstruction, single bundle repair, double bundle repair

## Abstract

Objective

To compare long-term functional results of ACL reconstruction with a single bundle (SB) and double bundle (DB).

Methods

Sixty patients who underwent ACL reconstructions from January 2007 to December 2008 were retrospectively evaluated (30 SB and 30 DB ACL reconstructions). Clinical and functional outcomes were measured pre- and postoperatively in terms of anterior drawer test, Lachman’s test, pivot shift test, KT1000 side-to-side difference, range of motion, International Knee Documentation Committee Scoring, Lysholm knee scoring scale (LKS), and Tegner activity level scale. The period of follow-up was 10 years.

Results

Clinical outcome measured showed that anterior drawer test result were equally normal for both groups (93.3%; p > 0.995); however, the Lachman test was 76.7% in the DB group and 56.7% in the SB group (p > 0.100), the pivot shift was 83% in the DB group and 50% in the SB group (p < 0.001), and KT1000 was 76.7% in the DB group and 56.7% in the SB group (p > 0.100). Regarding the functional outcome, it favored the DB group of patients, with the LKS being statistically significant (p < 0.007) and the Tegner activity level scale p-value being <0.001

Conclusions

DB ACL reconstruction produces better rotational stability and gives superior functional outcome in terms of return to pre-injury activity level in comparison to SB reconstruction. DB ACL reconstruction using hamstring tendon autograft produces better functional results at 10 years follow-up.

## Introduction

Anatomical observation has shown that the anterior cruciate ligament (ACL) mainly consists of two distinct bundles, the anteromedial (AM) bundle and the posterolateral (PL) bundle [1]. Conventional single-bundle (SB) ACL reconstruction techniques have focused on the restoration of the AM bundle while giving limited attention to the PL bundle. However, biomechanical studies show increased anterior and rotational stability with double-bundle (DB) compared to SB ACL reconstruction. The controversy remains in which surgery technique and length of the graft should be used [2]. The purpose of this study is to compare the outcomes of ACL reconstruction patients when using either DB or SB technique with similar rehabilitation in both techniques.

## Materials and methods

This was a retrospective non-randomized comparative study that was carried out at the Orthopaedic Arthroscopic Sports Unit, Hospital Kuala Lumpur (HKL). This study included all patients admitted to HKL and underwent either anatomical DB or SB ACL reconstructive surgery from January 2007 to December 2008. The study population was adults over 18 years and below 40, including both genders. Selected patients had pre-operative findings as follows: anterior drawer test > 3 mm, Lachman test > 3 mm, pivot shift test + (glide), KT 1000 > 3 mm, and functional hop test < 90%. Patients who underwent primary unilateral ‘anatomical’ single (AM) bundle or ‘anatomical’ double (AM and PL) bundle reconstructions of the ACL using only hamstring tendon autograft were also included in this study. The surgery was performed by several qualified Orthopaedic surgeons using the same technique. Furthermore, we also included patients who underwent the same post-ACL reconstruction rehabilitation therapy, which includes pain and swelling control, restoration of the normal range of motion, and development of muscle strength.

Patients who had a revision ACL and those with concomitant ipsilateral ligamentous injury were excluded from this study. Furthermore, patients with contralateral ACL-deficient or reconstructed knee, those who had a history of any ligament injuries in the contralateral knee, and those who underwent subtotal or total meniscectomy or meniscus repair for meniscus injury were also excluded from this study to avoid confounding factors.

All the patients were assessed both clinically and functionally pre- and post-surgery. In terms of clinical assessment, all patients were examined using the anterior drawer test at 90 degrees of flexion, Lachman test at 25 degrees of flexion, pivot shift test, KT1000 (side-to-side differences) at 25 degrees of flexion, and range of motion. The functional outcome was measured from two years of the ACL reconstruction until 10 years follow up and was further evaluated using the International Knee Documentation Committee (IKDC), Lysholm knee scoring scale (LKS), and Tegner activity level scale.

The anterior drawer test and Lachman test were further divided into normal and nearly normal, whereby knee laxity less than 3 mm is normal and knee laxity less than 5 mm is nearly normal for both tests. The study was analyzed by SPSS Version 25.0 (BM Corp., Armonk, NY, USA) using the chi-square test and t-test method.

## Results

There were 60 patients recruited throughout the study. Among them, 49 were males and 11 were females. Equal numbers of patients were treated with both types of the bundle. Among them, 30 received SB treatment and the remaining received DB reconstruction. The mean (SD) of age was 26.62 (6.727) years. All the patients had either normal or nearly normal outcomes. The data are summarized in Table 1.

**Table 1:** Baseline characteristics

Variables	N (%)
Age, mean (SD)	26.62 (6.727)
Gender, n (%)	
Male	49 (23.1)
Female	14 (35.9)
Bundle, n (%)	
Single	30 (50.0)
Double	30 (50.0)
Test results, n (%)	
Anterior drawer test	
Normal	56 (93.3)
Nearly normal	04 (06.7)
Abnormal	0
Severe abnormal	0
Lachman test	
Normal	40 (66.7)
Nearly normal	20 (33.3)
Abnormal	0
Severe abnormal	0
Pivot shift test	
Normal	45 (75.0)
Nearly normal	15 (25.0)
Abnormal	0
Severe abnormal	0
Functional hop test	
Normal	36 (60.0)
Nearly normal	24 (40.0)
Abnormal	0
Severe abnormal	0
KT1000­_n, n (%)	
Normal	40 (66.7)
Nearly normal	20 (33.3)
Abnormal	0
Severe abnormal	0

The association between test and type of bundles is shown in Table 2. There was no association between gender (female, male) and the type of bundle (SB and DB). The treatment outcome was not affected by the gender. There was no association between anterior drawer test and the type of bundles (SB and DB). The number of patients having normal result of SB is the same as that of DB. The same goes with Lachman test between the bundles. No significant association was found.

**Table 2:** Association between test and type of bundles ^a^Pearson’s chi-square test for independence. ^b^Fisher’s exact test.

Variable	Single bundle	Double bundle	X^2^ statistics (df)	p-Value^a^
Gender, n (%)				
Male	22 (73.3)	27 (90.0)		0.095
Female	8 (26.7)	3 (10.0)		
Anterior drawer test, n (%)				
Normal	28 (93.3)	28 (93.3)		>0.995^b^
Nearly normal	2 (06.7)	2 (06.7)		
Lachman, n (%)				
Normal	17 (56.7)	23 (76.7)		0.100
Nearly normal	13 (43.4)	7 (23.3)		
Pivot shift, n (%)				
Normal	15 (50.0)	25 (83.0)		<0.001^b^
Nearly normal	15 (50.0)	5 (17.0)		
Functional hop test, n (%)				
Normal	12 (40.0)	24 (80.0)		0.002
Nearly normal	18 (60.0)	6 (20.0)		
KT 1000_n, n (%)				
Normal	17 (56.7)	23 (76.7)		0.100
Nearly normal	13 (43.4)	7 (23.3)		

Comparison means of knee range of motion and LKS_n between types of bundles is shown in Table 3. There was no statistically significant difference in knee range of motion between SB and DB. However, in LKS_n, there was a statistically significant difference (p = 0.007) between SB and DB. The LKS_n mean and standard deviation in DB was a bit higher (93.50 and 4.26, respectively) than that in SB (89.0 and 2.58, respectively).

**Table 3:** Comparing means of knee ROM and Lysholm Knee Scoring Scale (LKS_n) between types of bundles ^a^Independent t-test ROM, range of motion

Variable	Single Bundle	Double Bundle	Mean difference (95% CI)	t statistics (df)	p-Value^a^
Knee ROM, mean (SD)	135.17 (4.82)	136.50 (4.58)	1.50 (-1.99, 4.99)	0.862 (58)	0.392
LKS_n, mean (SD)	89.0 (2.58)	93.50 (4.26)	3.70 (1.07, 6.33)	2.814 (58)	0.007

Both pre-Tegner and post-Tegner scores show a statistically significant in SB and DB treatment, with a p-value of < 0.001. Both bundles show a decrease in the post-Tegner score. SB has mean (standard deviation) of 5.13 (1.28) post-Tegner scoring, whereas the mean (standard deviation) of DB post-Tegner scoring was 16.46 (2.96), as shown in Table 4.

**Table 4:** Change of Tegner measurement within the type of bundle ^a^Paired t-test

Variable	Pre-Tegner score	Post-Tegner score	Mean difference (95% CI)	t statistics (df)	p-Value^a^
Bundle, mean (SD)					
Single	6.30 (1.29)	5.13 (1.28)	1.17 (0.87, 1.46)	8.074 (29)	<0.001
Double	18.53 (2.59)	16.46 (2.96)	0.80 (0.59, 1.01)	7.954 (29)	<0.001

## Discussion

Currently, there are many studies focusing on determining if DB ACL reconstruction is superior to SB reconstruction, but only a minority of them present a high level of evidence. Systematic reviews of appropriate studies are often the best form of evidence-based data, and reviews of level I and II studies constitute the highest level of evidence. However, results and conclusions from a randomized controlled trial are not always reliable, as such a trial can be performed and reported with methodical errors in current orthopedic and sports traumatology literature.

In this study, SB and DB surgical techniques were compared to determine differences in both the clinical assessment and functional outcome in patients with isolated rupture of the ACL before and after reconstructive surgery. The goals of anatomical ACL reconstruction are to restore 80-90% of the native ACL anatomy and to maintain long-term knee health [3]. Experience with SB ACL reconstruction has shown that it is as successful as DB but inadequately restoring anterior-posterior (A-P) stability [4].

As in our study, at the end of 10 years of follow-up, the results showed that the DB procedure did not yield better anterior drawer stability in patients undergoing the SB procedure in terms of statistical significance (p > 0.995); nevertheless, both procedures did produce a clinical significance when compared to pre- and post-operative ACL reconstruction (SB: 28 normal and 2 near normal; DB: 28 normal and 2 near normal). In regards to the Lachman test, both groups did provide better clinical outcomes in terms of patient number (SB: 17 normal and 13 near normal; DB: 23 normal and 7 near normal); however, the statistical differences were insignificant (p < 0.100).

We also found that side-to-side KT1000 arthrometer measurements were not statistically significant (p > 0.100) when compared between SB ACL reconstruction and DB ACL reconstruction. Our interpretation of lack of clinical significance is based on the following considerations: (1) the KT1000 arthrometer measures anterior knee laxity in 1-mm (as opposed to smaller) increments of precision, (2) the original description of instrumented measurement of anterior knee laxity using the KT1000 arthrometer reports a mean of 0.8 mm, standard deviation of 0.7 mm, and normal knee side-to-side laxity of greater than 0.52 mm, and (3) the IKDC considers a postoperative side-to-side difference of up to 2 mm as normal.

However, subjective symptoms of ‘giving-way’ and a positive pivot shift test revealed that rotational laxity often remained in the SB reconstructed knee. This is because the PL bundle, which is not traditionally reconstructed, plays a significant role in rotatory stability in the knee. From this study, the DB reconstruction did significantly reduce rotational instability. There were both clinical (SB: 15 normal and 15 near normal; DB: 25 normal and 5 near normal) and statistical significance (p < 0.001) with regard to the pivot shift examination. Clinical experience has suggested that biomechanical considerations of A-P translation alone do not correlate with subjective evaluations of knee stability and that a more complete evaluation of the role of rotational stability is relevant.

Therefore, in recent years, closer attention has been given to the rotational stabilizing function of the ACL. Included in the cadaveric study of 10 knees by Gabriel et al. was an analysis of a combined rotatory load of 10 Nm valgus and 5 Nm internal tibial torque at 15° and 30° of flexion [5]. For the PL bundle, an in situ force of 21 N was recorded at 15° and 14 N at 30°. For the AM bundle, the in situ forces were 30 N and 35 N, respectively. This shows that both the AM and PL bundles contribute to the rotational stability of the knee at these angles.

Tashman et al. in an in vivo study that used dynamic dual-video fluoroscopy to evaluate the kinematics of the knee during walking and running on a treadmill in patients who underwent traditional SB reconstruction [6]. Specifically, SB ACL reconstruction restored normal anterior/posterior translation, but the knee was externally rotated by an average of 4° and adducted by an average of 3° compared to the contralateral normal knee. Although the magnitude of the abnormal rotations may seem small, the difference in external rotation is sufficient to move the contact point of the lateral tibial plateau 3.5 mm posteriorly, and the difference in adduction would decrease the medial joint space by 1.3 mm in an average-sized knee.

A meta-analysis of four randomized clinical trials by Meredick et al. revealed that although DB ACL reconstruction resulted in an insignificantly side-to-side difference in tibial translation, as measured with the KT1000, 88% of patients who underwent DB ACL reconstruction had a normal pivot shift test after surgery compared to 62% of those who underwent SB reconstruction [7].

Various studies by from Markolf et al., who compared clinical outcomes between DB ACL reconstruction and SB ACL reconstruction, have demonstrated that DB ACL reconstruction resulted in significantly better side-to-side differences in anterior translation and a significantly higher proportion of normal pivot shift tests [8-10].

Experience with SB ACL reconstruction has shown that it is successful in allowing most patients to return to activities and sports following surgery. However, meta-analyses by Freedman et al. in 2003 and Laxdal et al. in 2005 showed that there is a subset of patients (10%-40%) who remain subjectively unstable and/or are unable to return fully to their activities or regain prior function [11,12]. Fithian et al. reported in 2005 that degenerative joint disease is associated with traditional SB ACL reconstructions in up to 90% at seven-year follow-up in some studies [13].

Published outcomes, both short- and mid-term, have been favorable with regard to return to activity in the DB reconstructed knee. Since 2008, the publication of clinical trials investigating SB and DB outcome have accumulated substantially [14]. In fact, a meta-analysis published in 2008 found four randomized controlled trials (level I evidence) and an additional five prospective and retrospective comparative studies (levels II and III) to assess differences in the outcome of SB and DB reconstructions; their findings showed that there were clinically and statistically significant differences in LKS, IKDC, and Tegner results between both surgical techniques [15]. As to follow suit, following our study, after two years, the LKS scores were significant for the DB groups, as there was a statistical difference (p = 0.007) between the two groups. The Tegner functional outcome scores were also superior in the DB group as compared to the SB group, with a p-value of <0.001 indicating a significant difference.

However, there are some limitations in this study, whereby there was a loss in numbers of patient during the follow-up. Other than that, patients were not compliance to the rehabilitation regime, which affects the recovery speed post-surgery. There was poor monitoring in the progress of the patients due to patient load in the rehabilitation clinic.

## Conclusions

The two main goals of DB ACL reconstruction are to restore the native biomechanics and anatomical parameters of the knee and to maintain long-term knee health. While DB ACL reconstruction has theoretical advantages, these may be negated by the added complexity of the surgical procedure. Current studies have shown a clinical advantage of the DB technique over the SB technique, but whether this will result in better functional outcomes, in the long run, is yet to be determined.

Nevertheless, DB literature has given ACL surgery a ‘rebirth,’ which has allowed us to critically analyze ACL anatomy and our surgical techniques, either through SB or DB reconstruction. Therefore, more long-term studies are necessary to determine whether the restoration of knee kinematics to a more physiological state is accompanied by any improvement in the development of osteoarthritis and improved knee functions.
